# PIDNET: Polar Transformation Based Implicit Disentanglement Network for Truncation Artifacts

**DOI:** 10.3390/e26020101

**Published:** 2024-01-24

**Authors:** Guang Li, Xinhai Huang, Xinyu Huang, Yuan Zong, Shouhua Luo

**Affiliations:** School of Biological Science and Medical Engineering, Southeast University, Nanjing 210096, China; 220214912@seu.edu.cn (X.H.); 220224413@seu.edu.cn (X.H.); xhzongyuan@seu.edu.cn (Y.Z.)

**Keywords:** CT imaging, truncation artifacts, unsupervised model, disentanglement

## Abstract

The interior problem, a persistent ill-posed challenge in CT imaging, gives rise to truncation artifacts capable of distorting CT values, thereby significantly impacting clinical diagnoses. Traditional methods have long struggled to effectively solve this issue until the advent of supervised models built on deep neural networks. However, supervised models are constrained by the need for paired data, limiting their practical application. Therefore, we propose a simple and efficient unsupervised method based on the Cycle-GAN framework. Introducing an implicit disentanglement strategy, we aim to separate truncation artifacts from content information. The separated artifact features serve as complementary constraints and the source of generating simulated paired data to enhance the training of the sub-network dedicated to removing truncation artifacts. Additionally, we incorporate polar transformation and an innovative constraint tailored specifically for truncation artifact features, further contributing to the effectiveness of our approach. Experiments conducted on multiple datasets demonstrate that our unsupervised network outperforms the traditional Cycle-GAN model significantly. When compared to state-of-the-art supervised models trained on paired datasets, our model achieves comparable visual results and closely aligns with quantitative evaluation metrics.

## 1. Introduction

Due to the ability to provide relatively high-quality and high-resolution images in a fast and cost-effective manner, X-ray computed tomography (CT) has become one of the most commonly used imaging tools in many fields. Compared to other mainstream imaging methods, the advantage of CT imaging is its high spatial resolution, but it comes with the drawback of ionizing radiation. To fully exploit the benefits of high resolution, contemporary CT imaging has been increasing its resolution levels. However, as the resolution increases, it brings forth the issue of reduced imaging field of view, leading to unintentional truncation of projection data. On the other hand, to mitigate ionizing radiation exposure, one of the better choices in practice is to conduct scans specific to the region of interest. This will induce intentional truncation of projection data. The direct consequence of projection data truncation results in truncation artifacts, often referred to as cupping artifacts. The cupping artifacts can significantly impair image quality and distort CT values.

The issue of image reconstruction from truncated projection data is known as an interior problem. An interior problem is an ill-posed problem arising from incomplete projection data, and hence, traditional analytical reconstruction algorithms like FBP cannot solve this problem. For a long time, it was believed that the solution to the interior problem is not unique, and researchers tried to use the projection extrapolation methods to mitigate cupping artifacts [1,2,3,4,5], although inaccurate extrapolation may lead to deviations in CT values of the reconstructed image [6]. With further exploration, researchers have discovered that the interior tomography can obtain accurate and stable solutions under certain specific conditions, such as when the region of interest (ROI) includes boundaries of the object or a prior information inside the ROI is known [7]. The representative approaches employ a back-projection of the derivative of projection data, and then a Hilbert transform along the desired direction in the image domain [8,9,10].The emergence of these methods effectively addresses a wide range of interior problems. However, when the ROI is entirely inside the scanned object and no prior knowledge in the ROI is available, all these methods are no longer applicable. In order to cater to a broader range of application scenarios, model-based iterative reconstruction (MBIR) methods have been developed. In these methods, iterative reconstruction and total variation (TV) [11] are combined to reduce truncation artifacts based on the assumption that the scanned object is piecewise constant. Although these methods can enhance artifact removal to some extent, it is challenging to completely eliminate artifacts in practical applications. Therefore, in specific application scenarios, introducing specific priors such as low-dose [12] or low-resolution priors [13] into the basic MBIR model can further improve the truncation artifact removal. In recent years, deep learning methods have been extensively employed across various aspects of CT imaging [14], including low-dose image processing [15,16,17], limited-angle CT imaging [18,19], and sparse-view reconstruction [20,21,22,23], achieving significant successes. Some deep learning based models [24,25] are successfully used to remove truncation artifacts. These methods improve the generalization performance of the network by using truncated differentiated backprojection (DBP) data instead of truncated filtered backprojection (FBP) data as input. At present, most of the existing deep learning based methods are based on supervised models, requiring pairs of CT images which entails two sets: one with cupping artifacts and the other without. Unfortunately, acquiring paired images in real applications is usually impossible, and researchers can only use simulated paired data to train the supervised models, which are then transferred to remove truncation artifacts in real scenarios. But, images acquired in real-world scenarios may significantly differ from simulated data in terms of anatomical structures and truncation ratios, leading to a notable performance drop when well-trained network models are transferred to real datasets. The emergence of unsupervised models offers a potential solution to address this issue. Currently, the representative unsupervised models are Cycle-GAN [26] and its variants [27]. Cycle-GAN employs consistency loss to address the challenge of lacking paired data. These models have demonstrated significant application potential in medical imaging domains, such as image denoising [28], super-resolution imaging [29,30,31], and metal artifact reduction [31,32,33]. To the best of our knowledge, there are very few effective unsupervised methods specifically designed for truncation artifact removal. Therefore, we aim to develop an efficient unsupervised model to reduce truncation artifacts in this paper. We are aware that the Cycle-GAN model performs excellently in domain translation tasks involving unpaired data, allowing it to transform images of one style into another. However, Cycle-GAN is an end-to-end network that lacks directed constraints guiding the generation of latent space, limiting the enhancement of network performance. To address this, we develop an implicit disentanglement network capable of intervening in latent space encoding for truncation artifact removal. The disentanglement concept is from the ADN method for metal artifact removal [27], which explicitly separates metal artifacts and structure information using multiple encoders. The disentanglement network we propose is an implicit disentanglement network, which can directly guide truncation artifacts and image content information to be distinguishable in the latent space and meanwhile significantly simplify the complexity of the network by reducing the number of encoders. Furthermore, we know that truncation artifacts are global artifacts, which are different from other common artifacts such as low-dose artifacts, metal artifacts that possess local features. To effectively distinguish truncation artifacts and content information in the latent space, the network should have the capability of capturing these global features. U-Net [34] has multiple pooling layers, which gives it a very large receptive field, making it widely used for global feature extraction. Therefore, U-Net becomes a candidate network for our task, which can be used to extract truncation artifacts with global features. In order to better capture the global features of truncation artifacts, we introduce a domain transformation mechanism to better extract these features. Given the quasi-circular nature of truncation artifacts, we incorporate polar coordinate transformation to convert the 2D cupping-shaped features to line-shaped features which are easier to extract. Through this process, we significantly alleviate the disentanglement network’s burden in extracting features, making it easier for the network to disentangle truncation artifacts and content information in the latent space.

The remainder of this paper is organized as follows. In Section 2, we introduce the methods and models. Section 3 presents the experiments and results. In Section 4, results and relevant issues are discussed. Finally, in Section 5, the conclusions are drawn.

## 2. Methods

Assuming that the domain of images with truncation artifacts is the set $D_{t}$, and the images without artifacts belong to the set *D*, a traditional supervised model based on the paired dataset PI=$\left\{\left(I^{t},J\right)|I^{t}\in D_{t},J\in D\right\}$ is to learn a mapping function $g:D_{t}\to D$. The approaches based on supervised models have been extensively approved to be significantly effective. However, in practical scenarios, obtaining paired training data is often unfeasible, significantly limiting the applicability of supervised models. To address this issue, we establish an unsupervised method in this study. In an unsupervised model, we only need an unpaired dataset NPI=$\left\{\left(I^{t},J\right)|I^{t}\in D_{t},J\in D\right\}$ based on which a mapping function $f:D_{t}\to D$ can be obtained through unsupervised learning. The essence of the methods for removing truncation artifacts based on deep learning models lies in how to effectively separate artifact features from content features. Supervised models can rely on paired data to efficiently learn to separate these two types of features. Nevertheless, unsupervised models, due to the lack of paired data, exhibit relatively weaker performance in guiding the network to separate these two features. To boost the performance of unsupervised methods in this regard, we propose an implicit disentanglement model based on Cycle-GAN to enhance the separation of content features and artifact features in the latent space. To fully exploit the features of cupping artifacts, we have introduced a domain transformation mechanism, whose purpose is to transform the cupping-shaped features into line-shaped features, thereby enabling the encoder to achieve more effective extraction of features. The implicit disentanglement network with this domain transformation mechanism further enhances the ability to separate artifacts from content information under unsupervised conditions. In the following subsections, we will provide a detailed description of the domain transformation and the implicit disentanglement network.

### 2.1. Domain Transformation

**Figure 1:**
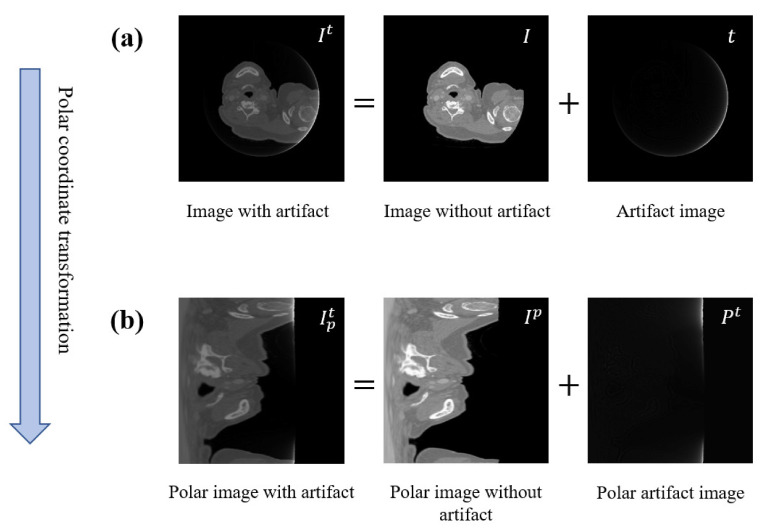
Preprocessing based on polar coordinate transformation. (**a**) shows the images without domain transformation, (**b**) represents the images after polar coordinate transformation.

Truncation artifacts present highly unique morphological characteristics on the reconstructed image. As shown in Figure 1a, truncation artifacts appear as cup-shaped structures, featuring bright circular borders that gradually diminish in intensity from the periphery towards the center. To enhance the extraction of the cup-shaped artifacts, we introduce a common domain transformation called the polar transformation as a preprocessing step before feeding the input image into the network. Assuming the center point $\left(C_{x},C_{y}\right)$ of the input image as the origin of the target polar coordinate system, we can transform any point (x,y) in the input image to its corresponding polar coordinates (ρ,ϕ). After this procedure, we obtain the transformed image $I_{p}^{t}\left(\rho ,\phi \right)$. Figure 1b illustrates the image after undergoing the polar coordinate transformation, revealing that the cup-shaped artifacts have been converted into line-shaped artifacts. Clearly, most of the artifacts present in this image can be removed using only basic horizontal local filters. Given the enhanced local filtering capabilities of deep neural networks, employing such networks to process images like this makes the separation of artifacts even more straightforward and more efficient.

### 2.2. Unsupervised Model Based on Implicit Disentanglement

**Figure 2:**
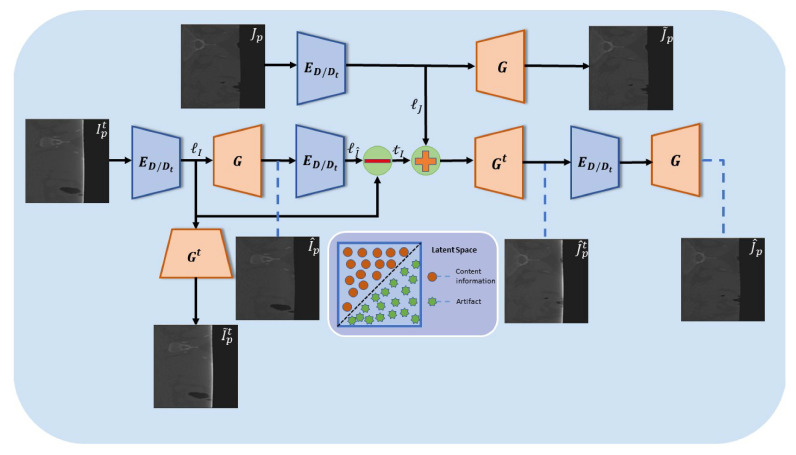
The overall architecture of the implicit disentanglement network for removing truncation artifacts.

The overall architecture of the implicit disentanglement network is shown in Figure 2. The input images of the network are $I_{p}^{t}$ and $J_{p}$, which are obtained by applying polar coordinate transformation to unpaired images $I^{t}$ and *J* from domains $D_{t}$ and *D*, respectively. The whole network consists of two pairs of encoder–decoder subnetworks: $\left\{E_{D/D_{t}},G\right\}$ for transforming images with artifacts into artifact-free images, and $\left\{E_{D/D_{t}},G^{t}\right\}$ for generating images with artifacts from artifact-free images. Notably, the subnetworks in our model shares the same encoder $E_{D/D_{t}}$, resulting in a simpler network structure compared to the traditional Cycle-GAN network that possess multiple encoders. We refer to this network as “implicitly disentangled” because we do not employ multiple encoders to explicitly extract image features and artifact features from the input images. Instead, we use a single encoder and attempt to concurrently construct an image feature subspace and an artifact feature subspace in the latent space. Moreover, we want these two subspaces to be separable for the decoders. With these design goals in mind, firstly, the image with cupping artifacts in polar coordinates $I_{p}^{t}$ is fed into the encoder, which maps $I_{p}^{t}$ to the latent space L, resulting in the feature information $\ell_{I}$. $\ell_{I}$ is composed of two parts: content features $c_{I}$ belong to the content space C, and the artifact features $t_{I}$ belong to the artifact space T, i.e., $\ell_{I}=c_{I}+t_{I}$. Next, content features $c_{I}$ are extracted through the decoder *G*, generating the artifact-removal image ${\hat{I}}_{p}$ in the polar coordinates. These two sub-procedures can be expressed as follows:

(1) $$\begin{array}{c} \ell_{I}=E_{D/D_{t}}\left(I_{p}^{t}\right), \end{array}$$

(2) $$\begin{array}{c} {\hat{I}}_{p}=G\left(\ell_{I}\right). \end{array}$$

Although the input in Equation (2) consists of the complete features $\ell_{I}$, the decoder *G* only extracts content features $c_{I}$. In other words, from the decoder’s perspective, the content and artifact features in the latent space are separable. To achieve this goal, we can impose a discriminative constraint on the output of the decoder first. However, due to the absence of a fidelity constraint, relying solely on the discriminative constraint is insufficient to guarantee the perfect extraction of the content feature. To better extract the content features, we also introduce the complementary constraint, which is the constraint for the artifact features since the artifact features can be regarded as the complementary features of the content features. Based on this logical relationship, we first separate the artifact features formally by introducing a short connection formulated as follows: complementary features of the content features. Based on this logical relationship, we first separate the artifact features formally by introducing a short connection formulated as follows:

(3) $$\begin{array}{c} t_{I}=\ell_{I}-\ell_{\hat{I}}=\ell_{I}-E_{D/D_{t}}\left({\hat{I}}_{p}\right). \end{array}$$

Then, we combine the artifact features $t_{I}$ and the mapping features $I_{J}$ which is obtained by mapping an artifact-free image $J_{p}$ into the latent space, and use a decoder $G^{t}$ that can extract both artifact and content features to generate a synthesized image with truncation artifacts,

(4) $$\begin{array}{c} {\hat{J}}_{p}^{t}=G^{t}\left(t_{I}+\ell_{J}\right). \end{array}$$

After that, we introduce a discriminative constraint on ${\hat{J}}_{p}^{t}$ to ensure a high degree of similarity between ${\hat{J}}_{p}^{t}$ and the real images with truncation artifacts. This encourages a better extraction of the artifact features $t_{I}$, meaning the extraction of content features is enhanced complementarily. At last, we reintroduce the previous encoder–decoder pair $\left\{E_{D/D_{t}},G\right\}$ into the network, and the artifact removal synthesized image is written as

(5) $$\begin{array}{c} {\hat{J}}_{p}=G\left(E_{D/D_{t}}\left({\hat{J}}_{p}^{t}\right)\right). \end{array}$$

In this process, the presence of a reference image as a constraint allows for better artifact truncation removal by the encoder–decoder pair $\left\{E_{D/D_{t}},G\right\}$. Furthermore, since this encoder–decoder pair constitutes the final artifact removal network we require, utilizing synthesized paired images can further enhance its capability of removing truncation artifacts under unsupervised conditions.

### 2.3. Learning Process

The core objective of this study is to establish an encoder $E_{D/D_{t}}$ capable of efficiently extracting content features under unsupervised conditions, along with a decoder *G* that can generate artifact-free images based on these features. However, in the absence of paired data, it is difficult to obtain an encoder–decoder pair that can efficiently separate content features while reducing truncation artifacts due to the lack of fidelity constraints. To address this issue, we need to introduce additional auxiliary subnetworks and supplementary constraints to reduce the reliance on fidelity constraints. We design five loss functions, including adversarial loss $L_{adv}$ which is the combination of adversarial content loss $L_{adv}^{D}$ and adversarial cupping artifact loss $L_{adv}^{D_{t}}$. Additionally, there are artifact consistency loss $L_{\mathrm{art}\;}$, self-reconstruction loss $L_{rec}$, cycle consistency loss $L_{cycle}$, and total variation loss $L_{tv}$. Therefore, the overall objective function is the weighted sum of these losses,

(6) $$\begin{array}{c} L=\lambda_{adv}\left(L_{adv}^{D}+L_{adv}^{D_{t}}\right)+\lambda_{\mathrm{art}\;}L_{\mathrm{art}\;}+\lambda_{\mathrm{rec}\;}L_{rec}+\lambda_{\mathrm{cycle}\;}L_{cycle}+\lambda_{tv}L_{tv}, \end{array}$$

where the λ are hyper-parameters to balance the importance of each loss.

**Adversarial loss**: If we want $E_{D/D_{t}}$ and *G* to perform well in the absence of paired images, the basic way is to introduce discriminators to guide the training. In this network, we introduce two discriminators. The first discriminator $D_{D}$ is located after the first decoder *G*. Its role is to discriminate whether an image comes from the network-generated images or from the truncation artifact-free image set. Training this discriminator allows the network-generated images to closely resemble truncation artifact-free images. The loss function based on this discriminator can be expressed as follows:

(7) $$\begin{array}{c} L_{adv}^{D}=E_{D}\left[\log D_{D}\left(J_{p}\right)\right]+E_{D_{t}}\left[1-\log D_{D}\left({\hat{I}}_{p}\right)\right] \end{array}.$$

Relying solely on the discriminator $D_{D}$ can make the generated image images and the truncation artifact-free images similar in terms of features, but it is still not sufficient to guarantee anatomical consistency. As mentioned above, we have decoupled the truncation artifact features implicitly, separating them from the anatomical features. Based on these separated truncation artifact features and truncation artifact-free images, we can generate synthesized truncation artifact-affected images. To encourage that the synthesized images are consistent with real truncation artifact-affected images in terms of features, we introduce another discriminator $D_{D_{t}}$ and related loss function as follows:

(8) $$\begin{array}{c} L_{adv}^{D_{t}}=E_{D_{t}}\left[\log D_{D_{t}}\left(I_{p}^{t}\right)\right]+E_{D,D_{t}}\left[1-\log D_{D_{t}}\left({\hat{J}}_{p}^{t}\right)\right]. \end{array}$$

Because promoting feature consistency between synthesized images and real artifact-affected images needs to extract full truncation artifact features in the latent space, the introduction of discriminator $D_{D_{t}}$ can indirectly promote separation of artifact features in the latent space. The objective function under the combined action of these two discriminators can be expressed as:

(9) $$\begin{array}{c} L_{adv}=L_{adv}^{D}+L_{adv}^{D_{t}}. \end{array}$$

**Reconstruction loss**: The encoder $E_{D/D_{t}}$ is used to encode truncation artifact-affected images and truncation artifact-free images. It maps content features to content feature space and artifact features to artifact feature space. Decoder $G^{t}$ is responsible for decoding all latent space features. To ensure that no intrinsic features are lost when input images pass through this encoder–decoder pathway, we establish the reconstruction loss as follows:

(10) $$\begin{array}{c} L_{\mathrm{rec}\;}=E_{D,D_{t}}\left[{∥{\tilde{I}}_{p}^{t}-I_{p}^{t}∥}_{1}+{∥{\tilde{J}}_{p}-J_{p}∥}_{1}\right]. \end{array}$$

The first term in the equation enforces that there is no feature loss when artifact-affected images pass through the $\left\{E_{D/D_{t}},G^{t}\right\}$ encoder–decoder pathway, while the second term ensures that there is no content feature loss when artifact-free images pass through the $\left\{E_{D/D_{t}},G\right\}$ pathway. In this way, we can consider that when the artifact-affected image passes through the $\left\{E_{D/D_{t}},G\right\}$ pathway, the content features are extracted. And, because when it passes through the $\left\{E_{D/D_{t}},G^{t}\right\}$ pathway all features including content and artifact features are extracted, we can approximately assume that the difference $t_{I}$ between the two pathways is the entirety of the artifact features.

**Artifact consistency loss**: In the adversarial loss, the discriminator $D_{D}$ is used to discriminate artifact-free images, allowing it to separate the artifact features. The promotion of the discriminator $D_{D_{t}}$ on discriminating synthetic artifact images can enable $D_{D}$ to more comprehensively separate the artifact features. This is because the promotion of $D_{D_{t}}$ would force the generator $G^{t}$ to generate more realistic synthesized artifact-affected images, thus causing more comprehensive artifact features to be passed by $t_{I}$. However, we know that a generative adversarial network has a powerful capability in generating data that matches the desired data distribution through adversarial learning. This might result in the incomplete decoding of artifact features passed by $t_{I}$, thereby, the discriminative performance improvement of $D_{D_{t}}$ may not fully reversely promote the encoding–decoding pair $\left\{E_{D/D_{t}},G\right\}$ to separate artifact features. To avoid this issue, we introduce the artifact consistency loss as follows,

(11) $$\begin{array}{c} L_{\mathrm{art}\;}=E_{D,D_{t}}\left[{∥\left(I_{p}^{t}-{\hat{I}}_{p}\right)-\left({\hat{J}}_{p}^{t}-J_{p}\right)∥}_{1}\right]. \end{array}$$

This ensures that the artifact features generated by $G^{t}$ originate from the separated artifact features, and ensures that the performance improvement of $D_{D_{t}}$ can effectively promote the extraction of artifact features by the encoding–decoding pair $\left\{E_{D/D_{t}},G\right\}$.

**Cycle consistency loss**: The ultimate goal of the network is to build an effective encoding–decoding pair $\left\{E_{D/D_{t}},G\right\}$ that can efficiently eliminate truncation artifacts. However, due to the lack of paired data, it is not possible to directly construct a regression model. Therefore, we utilize the separated artifact features $t_{I}$ and artifact-free image $J_{p}$ to generate simulated artifact-affected image ${\hat{J}}_{p}^{t}$. This allows us to create synthetic paired data $\left\{{\hat{J}}_{p}^{t},J_{p}\right\}$, thus indirectly constructing the following regression model to train the encoding–decoding pair $\left\{E_{D/D_{t}},G\right\}$

(12) $$\begin{array}{c} L_{\mathrm{cycle}\;}=E_{D,D_{t}}\left[{∥{\hat{J}}_{p}-J_{p}∥}_{1}\right]. \end{array}$$

**Total variation loss**: Truncation artifacts are cup-like artifacts with the following characteristics: the pixel intensity gradually decreases from the edge to the center in a radial direction, and the intensity change is a smooth transition. In the original images, it is challenging to directly construct a regularizer to constrain this feature. In the images after polar transformation, the radially decreasing artifacts are transformed into a horizontally decreasing artifacts, making it easier to establish constraints on this feature. Here, we introduce a horizontal total variation term to constrain the truncation artifacts as follows,

(13) $$\begin{array}{c} L_{tv}=\int_{\Omega }\sqrt{u_{x}^{2}}dx, \end{array}$$

where Ω is the support domain of the difference image $I_{p}^{t}-{\hat{I}}_{p},u_{x}=\frac{\partial u}{\partial x}$.

### 2.4. Network Details

**Figure 3:**
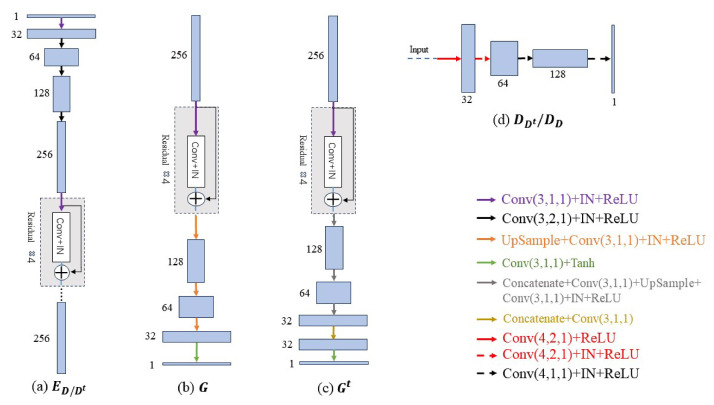
The detailed network architectures of the primary parts in PIDNET; (**a**) shows the details of the encoder $E_{D/D_{t}}$, (**b**) is the generator *G*, (**c**) represents the generator $G^{t}$, and (**d**) denotes the discriminator, $D_{D_{t}}/D_{D}$. IN refers to Instance Normalization, while ReLU represents LeakyReLU, except for $E_{D/D_{t}}$. Additionally, *k*, *s*, *p* in Conv(k,s,p) signify the kernel size, stride, and padding size, respectively.

The whole network consists primarily of five parts: one encoder, two decoders, and two discriminators. The detailed network architectures of these components are shown in Figure 3. The encoder subnetwork and the two decoder subnetworks form two encoder–decoder pairs. The encoder subnetwork consists of five convolutional layers, of which only three have down-sampling operations, since extracting the truncation artifact features in polar coordinates does not require a very large receptive field. The structures of the two decoders correspond to that of the encoder, so each decoder subnetwork is also composed of five convolutional layers, employing three up-sampling operations to restore the image to its original size. The two discriminators share the same network architecture. Because many studies such as CycleGAN [27] and Pix2Pix [35] have already demonstrated that the PatchGAN discriminator is more advantageous for style transfer tasks compared to the general GAN discriminator, we also adopt the PatchGAN discriminator in this study.

## 3. Experiments and Results

### 3.1. Dataset

#### 3.1.1. Clinical Dataset

In our experiment, we prepared two clinical datasets named CL1 and CL2. These datasets originated from the American Association of Physicists in Medicine (AAPM) and the National Cancer Institute’s Cancer Imaging Archive (TCIA), respectively. In dataset CL1, there were a total of 11,924 pairs of paired chest CT images. When training the supervised models, we selected 10,000 pairs forming the training set and the remaining 1924 pairs forming the test set. When training the unsupervised model, the artifact-affected images in the training dataset formed the set $D_{t}$, while the artifact-free images formed the set *D*. To enable a direct comparison of the supervised and unsupervised model performance in the absence of paired data and evaluate the performance change of supervised models transferred to similar datasets, on dataset CL2, we used 2427 pelvis CT images with truncation artifacts to form the set $D_{t}$, and 2427 artifact-free CT images to form the set *D*. Additionally, we used 300 pairs of paired images to form the test set. The image size for both datasets CL1 and CL2 was 384×384. In the simulated fan-beam projection, the number of projection views was set to 720, and the data truncation proportion was set to 30%. The reconstruction method employed was FBP.

#### 3.1.2. Preclinical Dataset

In order to illustrate the decline in performance metrics of traditional supervised models when transferred to datasets with exacerbated feature disparities from the original training set and simultaneously validate the advantage of our proposed PIDNET, we constructed a preclinical dataset derived from our self-developed micro-CT system [36,37]. We obtained projection data from multiple mice and reconstructed 2752 CT images using the FDK method. Compared to dataset CL2, these images exhibit a greater disparity from the clinical dataset CL1. While our proposed PIDNET model is applicable in cone-beam CT, it is important to note that the classical comparison method, E2E U-Net, is exclusively compatible with fan-beam CT. Additionally, due to the memory constraint, applying the classical TV method to cone-beam CT is not straightforward. Hence, we projected the acquired CT images using fan-beam geometry and reconstructed them utilizing the FBP method. The truncation proportion in the experiment was set to 30%. We utilized 2452 artifact-affected images to form the dataset $D_{t}$ and 2452 artifact-free images to form the dataset *D*. Additionally, 300 pairs of paired data constituted the test set.

### 3.2. Evaluation Metrics

To comprehensively evaluate the proposed method, we used two evaluation metrics to measure the performance of different methods in removing truncation artifacts, including Peak Signal-to-Noise Ratio (*PSNR*) and Structural Similarity Index (*SSIM*). *PSNR* is a commonly used metric to assess the quality of processed images, and it is described as follows:

(14) $$\begin{array}{c} PSNR\left(f^{gt},f^{p}\right)=20\times \log_{10}\left(\frac{NM{∥f^{gt}∥}_{\infty }}{{∥f^{gt}-f^{p}∥}_{2}}\right), \end{array}$$

where $f^{gt}$ represents the ground truth, $f^{p}$ represents the estimated image, and *M* and *N* are the number of rows and columns of the image pixels. *SSIM* is a metric used to measure the similarity between two images, and higher *SSIM* values indicate greater similarity and better image quality. *SSIM* is considered to be more in line with human perception of image quality compared to *PSNR*. The formula for *SSIM* is as follows:

(15) $$\begin{array}{c} SSIM\left(f^{gt},f^{p}\right)=\frac{\left(2\mu_{f^{gt}}\mu_{f^{p}}+c_{1}\right)\left(2\sigma_{f^{gt},f^{p}}+c_{2}\right)}{\left(\mu_{f^{gt}}^{2}+\mu_{f^{p}}^{2}+c_{1}\right)\left(\sigma_{f^{gt}}^{2}+\sigma_{f^{p}}^{2}+c_{2}\right)}, \end{array}$$

where $\mu_{f^{gt}}$ and $\mu_{f^{p}}$ are the means of the ground truth and estimated images, and $\sigma_{f^{gt}}$ and $\sigma_{f^{p}}$ are the variances of the ground truth and estimated images, respectively. $\sigma_{f^{gt},f^{p}}$ represents the covariance between $f^{gt}$ and $f^{p}$. $c_{1}$ and $c_{2}$ are two stability constants used for division. We set them as $c_{1}={\left(k_{1}L\right)}^{2}$ and $c_{2}={\left(k_{2}L\right)}^{2}$, where *L* is the maximum pixel value of the image, and $k_{1}$ and $k_{2}$ are constant values set to 0.03.

### 3.3. Training and Testing

To provide a more objective evaluation of our method, we compared it with five other classic truncation artifact removal methods. Two of these methods are traditional models, including the extrapolation method and the iterative reconstruction method based on total variation constraint. For simplicity, they are referred to as Extrapolation and TV. Two others are state-of-the-art supervised models, namely U-Net [34] and the dual-domain model E2E U-Net [38]. The final one is the most classic unsupervised method, Cycle-GAN, widely used for unsupervised image translation. For U-Net and Cycle-GAN, we utilized their official code, while for the other three methods, we re-implemented the code based on the corresponding publicly published research papers. The proposed network was implemented using the PyTorch(2.0.0) deep learning framework. In terms of optimization, we employed the Adam optimizer with a learning rate of $1\times 10^{-4}$ to minimize the objective function. The hyper-parameters in the objective function were set as follows: $L_{adv}^{D}=L_{adv}^{D_{t}}=1.0,\;\lambda_{art}=\lambda_{rec}=5.0,\;\lambda_{cycle}=10.0,\;\lambda_{tv}=100.0.$

In our experiments, all supervised methods were trained on CL1 and directly transferred to other datasets because only CL1 had paired training data. However, all unsupervised methods, including the comparative unsupervised method, were trained and tested separately on the three datasets since they do not require paired data.

### 3.4. Results

In the experiment, we tested the method proposed in this paper and all the comparison methods on the clinical datasets CL1 and CL2 and the preclinical dataset. Quantitative analysis was conducted based on the evaluation metrics *SSIM* and *PSNR*.

**Figure 4:**
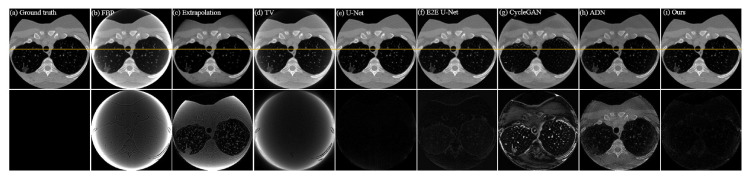
Qualitative comparison of different methods on dataset CL1; (**a**–**i**) represent the ground truth and the resulting images from FBP, Extrapolation, TV, U-Net, E2E U-Net, Cycle-GAN, ADN, and our PIDNET, respectively. The second row shows the residual images derived from comparing the resulting images with ground truth. The yellow lines indicate the positions of the profile plots shown in Figure 5.

**Figure 5:**
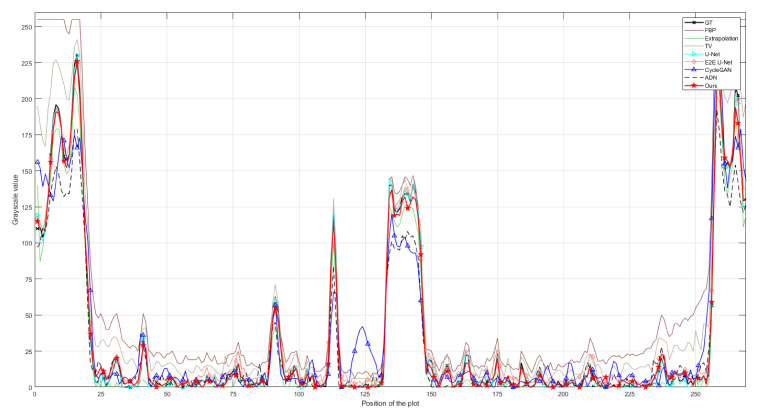
Comparison of the profile plots of the yellow lines marked in the resulting images shown in Figure 4.

The quantitative analysis results of all methods on the CL1 dataset are shown in Table 1. It can be intuitively observed from the table that, compared to traditional Extrapolation and TV methods, deep learning methods show a significant improvement in removing truncation artifacts. Among supervised models, E2E U-Net stands out in terms of metrics. Supervised models, particularly E2E U-Net, outperform the classical unsupervised model Cycle-GAN and the later improved unsupervised model ADN in performance metrics, mainly due to strong pixel-level fidelity constraints in supervised models. Looking at the performance metrics, our proposed unsupervised method shows a clear improvement over Cycle-GAN and ADN, approaching a similar performance to supervised models, especially with almost no difference in *SSIM* and only a slight disadvantage in *PSNR*. Figure 4 illustrates the result images of various comparison methods and the residual images compared to the ground truth. It is evident that Extrapolation and TV methods already exhibit significant improvement in removing truncation artifacts compared to the reconstruction image from FBP. However, there are still noticeable truncation artifacts left. Moreover, the Extrapolation method fails to recover many texture details. The two supervised models, U-Net and E2E U-Net, show substantial improvement over the traditional Extrapolation and TV methods in artifact removal performance. Residual images hardly exhibit noticeable truncation artifacts, and the unrecovered image details are minimal, making it challenging to observe differences from the ground truth image. Although the classical Cycle-GAN model may not completely restore texture details, it performs better than traditional Extrapolation and TV methods in truncation artifact removal, resulting in a significant reduction in truncation artifacts in the residual images. ADN performs better than Cycle-GAN in removing artifacts, but still falls short in restoring fine details. From the residual images, it is apparent that our proposed unsupervised method performs exceptionally well, achieving performance comparable to supervised models in artifact removal. Truncation artifacts are nearly indiscernible in the residual images, and unrecovered texture details are minimal. These results align with the quantitative analysis presented in Table 1. To visually demonstrate the consistency between the artifact-removed images and the ground-truth image, we provide profile plots along the orange lines indicated in Figure 4. These plots are shown in Figure 5. From this figure, we can see that the profile plot from our unsupervised model and the plots from the competing supervised models are close to each other, and meanwhile, they closely align with the ground-truth profile plot.

**Figure 6:**
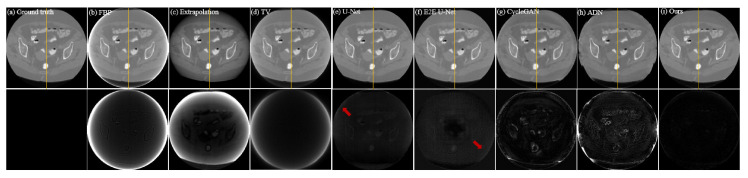
Qualitative comparison of different methods on dataset CL2; (**a**–**i**) represent the ground truth and the resulting images from FBP, Extrapolation, TV, U-Net, E2E U-Net, Cycle-GAN, ADN, and our PIDNET, respectively. The second row shows the residual images derived from comparing the resulting images with ground truth. The yellow lines indicate the positions of the profile plots shown in Figure 7.

**Figure 7:**
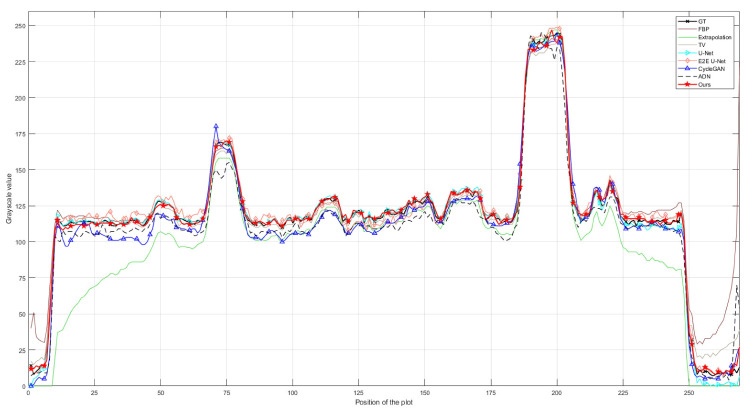
Comparison of the profile plots of the yellow lines indicated in the resulting images shown in Figure 6.

To simulate unsupervised scenarios, we only designed unpaired data in dataset CL2. Therefore, it was impossible to train supervised models on this dataset and the supervised models tested on dataset CL2 were directly transferred from dataset CL1. As both datasets CL1 and CL2 consist of clinical CT images, their similarity is relatively high. Testing the pre-trained models on dataset CL2 can be used to intuitively demonstrate the performance variation of supervised models when they are transferred between similar datasets. Table 2 displays the performance metrics of all comparative methods on dataset CL2. It is evident that the supervised models, being directly transferred from dataset CL1, exhibit a significant decline in all performance metrics. However, the final outcomes still surpass the traditional Extrapolation and TV methods, indicating the practical value of directly transferring pre-trained models. Table 2 also clearly shows that the performance metrics of our proposed unsupervised model are superior to the supervised models. This is because our model is unsupervised and can be trained without paired data. The results and residual images shown in Figure 6 support the same conclusion as Table 2. The supervised models outperform the traditional Extrapolation and TV methods, but their residuals are larger than those obtained on dataset CL1, indicating that more truncation artifacts (highlighted by red arrows) are not removed. Additionally, the comparison results of the profile plots in Figure 7 clearly demonstrate the superiority of the supervised models over traditional methods, while the profile plot from our unsupervised model is closer to the ground-truth profile plot than all comparison methods.

**Figure 8:**
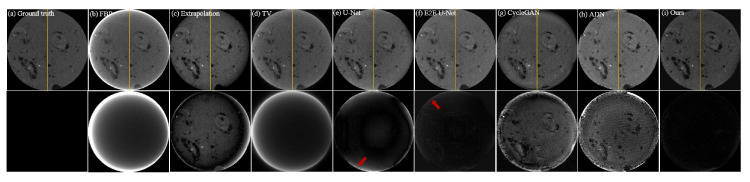
Qualitative comparison of different methods on preclinical dataset; (**a**–**i**) represent the ground truth and the resulting images from FBP, Extrapolation, TV, U-Net, E2E U-Net, Cycle-GAN, ADN, and our PIDNET, respectively. The second row shows the residual images derived from comparing the resulting images with ground truth. The yellow lines indicate the positions of the profile plots shown in Figure 9.

**Figure 9:**
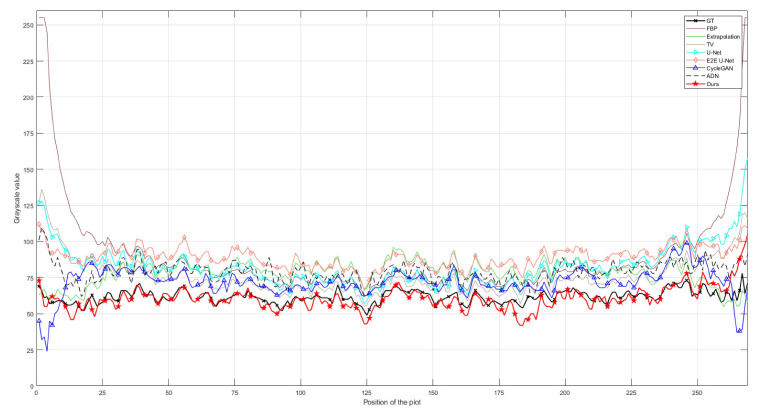
Comparison of the profile plots of the yellow lines indicated in the resulting images shown in Figure 8.

Because the preclinical data are derived from micro-CT, their similarity with dataset CL1 is lower compared to dataset CL2. Thus, we can simulate the performance changes of supervised models transferred to datasets with lower similarity and assess the performance advantage of unsupervised models. Table 3 presents the performance metrics of all comparative methods on this dataset. From this table, it is evident that the performance of supervised models in terms of *SSIM* and *PSNR* metrics continues to decline compared to the results on Dataset CL2, although their values are still better than traditional extrapolation and TV methods. This further illustrates that the performance of supervised models worsens with exacerbated changes in data characteristics. Conversely, our unsupervised model maintains good performance on this dataset. The results and residual images in Figure 8 distinctly show that our model is closest to the ground-truth images. The comparison of profile plots in Figure 9 demonstrates that our unsupervised method achieves the highest similarity with the ground-truth profile plot compared to other methods, which exhibit visible gaps from the ground-truth profile plot. Both qualitative and quantitative analyses indicate that our unsupervised model achieves the best performance in removing truncation artifacts on this dataset.

### 3.5. Ablation Study

#### 3.5.1. Performance of Model Components

To validate the effectiveness of the different components in our proposed model, six ablation experiments were conducted on dataset CL1. The configurations of the experiments are shown as follows:

Var1: only the adversarial loss $L_{adv}$ is introduced in the network.
Var2: the reconstruction loss $L_{rec}$ is added to Var1.
Var3: the artifact consistency loss $L_{art}$ is introduced to Var2.
Var4: the cycle consistency loss $L_{cycle}$ is added on the basis of Var3.
Var5: the polar coordinate transformation is incorporated into Var4.
Var6: the total variation loss $L_{tv}$ is added to Var5, and this is the final network configuration.

**Figure 10:**
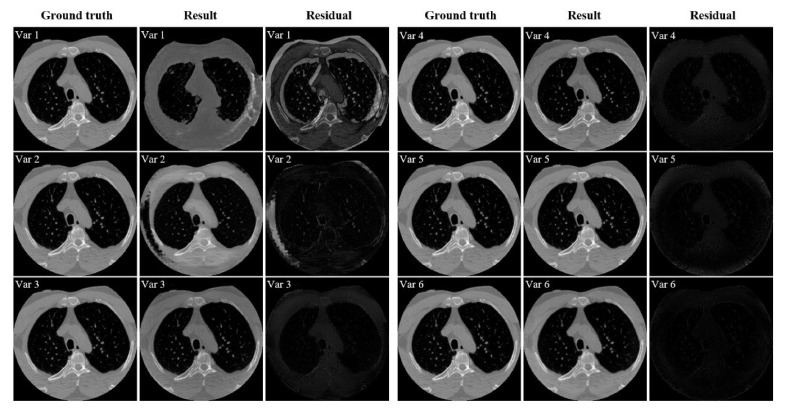
Qualitative analysis of the ablation study of the proposed PIDNET conducted on dataset CL1. Var1–Var6 mean different components added to the network. Each group’s three columns represent ground truth, resulting image, and residual image, respectively.

Table 4 shows a significant increase in *SSIM* and *PSNR* scores from Var1 to Var6. Var1, having only discriminative constraints, fails to recover many image details. However, when introducing the reconstruction loss in Var2, the network’s performance notably improves. At this stage, the network’s performance surpasses that of the classical unsupervised Cycle-GAN model. Subsequently, with the inclusion of artifact consistency and cycle consistency losses, the network’s performance sees a marked enhancement over Var2. *SSIM* increases by nearly 5 percentage points, and *PSNR* improves by approximately 4.6 dB. To further optimize the network, the polar coordinate transformation is incorporated in Var4, forming Var5. The performance metrics of Var5 show further improvement, indicating that polar coordinates indeed help enhance the separation of artifact features and content features. Finally, with the addition of lateral TV constraints in Var6, compared to Var4, *SSIM* increases by 1.6 percentage points, and *PSNR* improves by nearly 4 dB. These results demonstrate the effectiveness of polar coordinates and the lateral TV constraint. Consistent with Table 4, the residual images from Var1 to Var6 in Figure 10 are getting smaller, confirming that all introduced constraints and tricks significantly contribute to improving network performance.

#### 3.5.2. Impact of Truncation Ratio and Image Size

**Figure 11:**
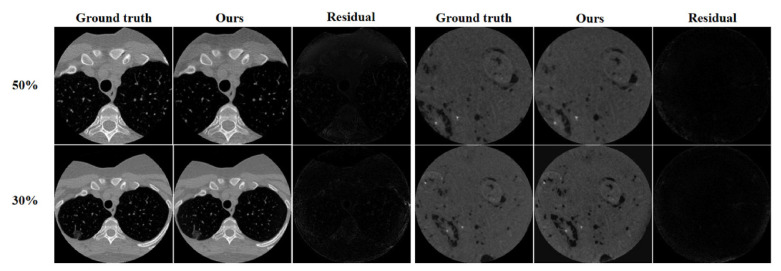
Comparison of results at different truncation ratios on dataset CL1 and preclinical dataset.

To validate the sensitivity of our proposed model to truncation ratios and image sizes, we conducted experiments using different truncation ratios and image sizes. In particular, we chose truncation ratios of 30% and 50%. After applying a 30% truncation ratio, the image size became 384 × 384, while a 50% truncation ratio resulted in an image size of 256 × 256.The results of the comparative experiments are shown in Figure 11.

From Figure 11, it can be observed that the resulting images and residual images under both truncation ratios and image sizes do not exhibit significant differences. Table 5 shows the *SSIM* and *PSNR* scores for the two experiments.The evaluation metrics at different truncation ratios do not differ much as the truncation ratio increases and the image size changes, which demonstrates that our method has good robustness and adaptability.

## 4. Discussion

The presence of truncation artifacts can significantly distort CT values, directly affecting the outcomes of image diagnosis. Moreover, the truncation issue remains a challenging problem in most application scenarios since it is an ill-posed problem. Although deep learning models have proven to effectively remove truncation artifacts in supervised settings where a large amount of paired data are needed. Nevertheless, obtaining paired data in numerous real application scenarios is impractical. Therefore, the conventional workflow for supervised models in practical applications is based on training with simulated paired data, followed by direct transfer to the target real dataset. In theory, the direct transfer strategy could degrade the performance of trained models depending on the similarity of the intrinsic features of the target dataset to the training dataset. In our experiments, we validated the performance degradation of the direct transfer models. Experimental results indicate that when the training dataset is transferred to a similar target dataset, the performance of truncation artifact removal decreases to some extent. As the dissimilarity between the target and training datasets increases, the performance degradation becomes more pronounced. This suggests that supervised models, while achieving excellent results during simulated training, face significant limitations in practical applications. Therefore, unsupervised models are essential in real applications. In this paper, we propose a novel unsupervised artifact removal model, which is an implicit disentanglement model. It introduces complementary constraints by implicitly disentangling the truncation artifacts, enhancing the performance of encoder–decoder pairs in removing truncation artifacts. The disentangled truncation artifact features also assist in generating simulated paired data more effectively, further strengthening the training of the encoder–decoder pair for truncation artifact removal. We also introduce other auxiliary strategies, such as polar coordinate transformation and the horizontal total variation strategy based on it. Experimental results show that the proposed unsupervised model achieves almost equivalent visual results as the state-of-the-art supervised models trained with paired data, with only a slightly lower *PSNR* score. In the absence of paired training data, the performance of the directly transferred supervised models degrades, whereas our proposed unsupervised model adapts well to this situation. Furthermore, the experiments confirm that our unsupervised model’s performance is far superior to the classical Cycle-GAN model and ADN, indicating that our proposed disentanglement model and the various auxiliary strategies effectively enhance the unsupervised model’s performance. Through qualitative and quantitative analyses in the ablation study, we can see that all introduced tricks contribute to some degree of improvement in the model. Specifically, various objective functions designed for the implicit disentanglement network greatly enhance the network’s performance, while the additional polar coordinate transformation and the horizontal total variation constraint based on this transformation further improve the network’s artifact removal performance. In this paper, we rely solely on the image domain to establish the unsupervised model, and we have demonstrated that the unsupervised model can achieve good performance in removing truncation artifacts and is comparable to the state-of-the-art supervised models. However, our network still has some limitations. First, there is room for improvement in restoring edge details. In future studies, we can enhance the performance of the model by integrating novel techniques, such as attention mechanisms [39,40], into our approach. Secondly, although our model is unsupervised, it still requires the support of non-truncated data with similar features. Achieving truncation artifact removal under the condition of only artifacted images available is the goal of our future research.Moreover, we know that in the research on various CT artifact removal, the combination of multiple domains such as the projection domain and image domain can be used to achieve better performance. Many previous studies have confirmed this point, so if unsupervised models combining with multiple domains are used to solve truncation artifact problem, there should be a better performance improvement. This will be our research focus in the future.

## 5. Conclusions

In this paper, we propose a simple and efficient unsupervised method for removing truncation artifacts. Our method is built upon the Cycle-GAN network, incorporating an implicit disentanglement model to separate truncation artifacts from content information. The separated artifact features are then utilized as complementary constraints to strengthen the training of the artifact removal subnetwork. Additionally, the method synthesizes simulated paired data using these artifact features, further enhancing the performance of the artifact removal subnetwork. Our method also introduces polar coordinate transformation and an innovative constraint based on this transformation, specifically designed for truncation artifact features. These innovative strategies significantly enhance the performance of the unsupervised network. Experiments conducted on multiple datasets demonstrate that the performance of this unsupervised network surpasses that of the classic Cycle-GAN model and ADN. Moreover, when compared to supervised models trained on paired datasets, our proposed model achieves almost equivalent visual results, closely resembling performance in *SSIM*, with only a slight disadvantage in *PSNR*. However, when transferring supervised models to scenarios without paired data, these models experience varying degrees of performance degradation. In contrast, the performance of our proposed unsupervised model remains unaffected. Both subjective and objective evaluation metrics confirm the superiority of our unsupervised model over classical supervised models. This indicates that unsupervised models have greater practical value in real-world applications.

## Figures and Tables

**Table 1:** Quantitative comparison of different methods in terms of PSNR(dB) and SSIM on dataset CL1.

		Method	Metrics	
			SSIM	PSNR
Chest	Conventional	Extrapolation	0.9143	21.0513
		TV	0.8210	24.9560
	Supervised	U-Net	0.9827	40.8604
		E2E U-Net	0.9897	42.9835
	Unsupervised	CycleGAN	0.8107	26.1695
		ADN	0.9666	32.0834
		Ours	0.9873	39.7273

**Table 2:** Quantitative comparison of different methods in terms of PSNR(dB) and SSIM on dataset CL2.

		Method	Metrics	
			SSIM	PSNR
Pelvis	Conventional	Extrapolation	0.8907	24.3274
		TV	0.8601	22.3092
	Supervised	U-Net	0.9763	35.7170
		E2E U-Net	0.9483	38.2473
	Unsupervised	CycleGAN	0.8899	28.1647
		ADN	0.9721	32.5045
		Ours	0.9947	42.4615

**Table 3:** Quantitative comparison of different methods in terms of PSNR(dB) and SSIM on the precinical dataset.

		Method	Metrics	
			SSIM	PSNR
Mouse	Conventional	Extrapolation	0.9082	25.3666
		TV	0.8465	20.5258
	Supervised	U-Net	0.9701	32.2261
		E2E U-Net	0.9257	35.9452
	Unsupervised	CycleGAN	0.8464	26.6343
		ADN	0.9702	32.1039
		Ours	0.9903	43.9631

**Table 4:** Quantitative analysis in the ablation study.

Model	Metrics	
	SSIM	PSNR
Var1 ($L_{adv}$ only)	0.7838	24.5578
Var2 (Var1 with $L_{rec}$)	0.9232	31.2989
Var3 (Var2 with $L_{art}$)	0.9573	33.7104
Var4 (Var3 with $L_{cycle}$)	0.9710	35.8856
Var5 (Var4 with polar transformation)	0.9805	37.5096
Var6 (Var5 with $L_{tv}$)	0.9873	39.7273

**Table 5:** Quantitative comparison of different truncation ratios in terms of PSNR(dB) and SSIM.

	SSIM		PSNR	
Ratio	30%	50%	30%	50%
Chest	0.9873	0.9885	39.7273	39.2435
Mouse	0.9903	0.9899	43.9631	42.3032

## Data Availability

CL1 and CL2 datasets are from the American Association of Physicists in Medicine (AAPM) and the National Cancer Institute’s Cancer Imaging Archive, respectively (TCIA). Preclinical dataset is not applicable.
